# Economic evaluation of interventions delivered by primary care providers to improve neurodevelopment in children aged under 5 years: protocol for a scoping review

**DOI:** 10.1186/s13643-017-0450-6

**Published:** 2017-03-21

**Authors:** Karen M. Edmond, Natalie A. Strobel, Kimberley McAuley, Elizabeth Geelhoed, Lisa Hurt

**Affiliations:** 1 School of Paediatrics and Child Health, The University of Western Australia, Perth, WA Australia; 2 School of Population Health, The University of Western Australia, Perth, WA Australia; 3 Division of Population Medicine, Cardiff University, Cardiff, UK; 4 School of Paediatrics and Child Health, The University of Western Australia, 35 Stirling Highway, Crawley, WA 6009 Australia

## Abstract

**Background:**

Frequently cited benefit-cost ratios suggest that interventions to improve neurodevelopment have high economic returns when implemented during pregnancy and early childhood. However, there are many challenges when primary care providers implement these interventions at scale, and it is unclear how many research studies or programmes have examined cost-effectiveness and which methods were used. There are no current scoping or systematic reviews which have assessed economic evaluations of interventions delivered by primary care providers to improve child neurodevelopment.

**Methods/design:**

The aim of this review is to describe the economic evaluations of interventions delivered by primary care providers to improve neurodevelopment in children aged 0–4 years. Specific subgroup analyses will include income level of country (high, middle and low); population type (universal vs targeted); time period when intervention was implemented (antenatal vs infancy [0–11 months] vs early childhood [12–59 months]); and setting (research study vs programmes evaluation at scale). All study designs will be included. The primary outcomes of interest are cost per neurodevelopmental or cognitive health gain in children aged 0–4 years. All measures of cost, neurodevelopment or cognitive function that have been previously validated as an appropriate test in this domain will be included. Databases such as MEDLINE (OVID), PsycINFO (OVID), EMBASE (OVID), CINAHL, Cochrane Library (including CENTRAL, DARE, HTA and NHS EED), Paediatric Economic Database Evaluation (PEDE) and WHO databases and reference lists of papers will be searched for relevant articles. Five phases will be followed: identifying the research question, identifying relevant studies, study selection, charting data and collating, summarising and reporting results. We will present cost and effectiveness data descriptively.

**Discussion:**

This review appears to be the first to be conducted in this area. The findings will be an important resource for future systematic reviews on interventions that have a cost component. This information will be valuable for policy makers and programmers who work in public health or primary care settings.

**Electronic supplementary material:**

The online version of this article (doi:10.1186/s13643-017-0450-6) contains supplementary material, which is available to authorized users.

## Introduction

Interventions delivered in early childhood have been shown to have substantial and sustained impacts on long-term cognitive and neurodevelopmental outcomes. Frequently cited benefit-cost ratios from the USA suggest that for every dollar invested in services for preschool age children, there will be a $2 to $2.60 return to society [7, 8]. The economic return has been estimated at between 15 and 17% for every dollar taking into account crime, education and welfare savings and increased taxes due to higher earnings [7, 8].

Primary care providers are health professionals who work at the first level of the health service and are trained in clinical care. They include community health workers, Indigenous health workers, generalist nurses, health visitors, midwives, child health nurses, general practitioners and other primary care doctors. Primary care providers routinely provide face to face ‘non-medicinal’ interventions such as advice, counselling and the promotion of behaviour change for their clients. Pregnant women, families and care givers of young children receive anticipatory guidance, health promotion, health education, promotional interviewing and motivational interviewing. They also receive screening, surveillance and ‘brief interventions’ (time-limited interventions implemented by primary care providers that focus on praising and reinforcing or changing caregiver behaviour). Delivery channels may include home visiting, clinic visits, group programmes, telehealth, antenatal care and child health checks. Examples include screening for postnatal depression and developmental milestones, the World Health Organization (WHO)/Unicef Care for Child Development package [20], the WHO Thinking Healthy programme [22] and the nurse family partnership model for high-risk families [15, 17]. Many of these programmes have been highly effective in improving children’s long-term neurodevelopmental outcomes when implemented in highly controlled research settings. However, there are many challenges when implementing these programmes at scale, and it is unclear how many research studies or programmes have examined cost-effectiveness and which methods were used.

There are also increasing numbers of alternate channels available for delivery of interventions to reinforce and change the behaviour of children’s care givers including electronic and social media (e.g. biofeedback, chat rooms, mass media, plain packaging, social marketing, websites, web boards and webinars). Face to face access to primary care providers can be limited especially in remote areas; their services can be expensive to maintain and their quality of care is variable. Reinforcing and changing behaviour is also difficult and requires a specific skill set, high quality training and a population who are ‘ready to change’. Indeed, it has been suggested that face to face encounters with mainstream primary care providers should be restricted to acute care and targeted screening and that behaviour change interventions should be provided by other channels [17].

Systematic reviews have assessed the effect of medicinal approaches to improving child neurodevelopment such as vaccinations [4] and nutritional supplementation including vitamin A [6] and iron [21]. ‘Non-medicinal’ systematic reviews have focused on interventions to promote access [14], combined impact of ‘packages’ of interventions [12], maternal mental health interventions [9, 16] and child weight management programmes [1]. However, no scoping reviews, inventories or narrative assessments of cost and cost-effectiveness appear to have been published. This information is important for health professionals to make policy decisions and implement programmes on a large scale.

### Objectives

The aim of this review is to ‘scope’ , describe and provide an inventory of the studies that have assessed the economics of interventions delivered by primary care providers to improve child neurodevelopment in children aged 0–4 years using scoping review methodology [2, 3, 10]. Specific subgroup analyses will include income level of country (high, middle and low); population type (universal vs targeted); time period when intervention was implemented (antenatal vs infancy [0–11 months] vs early childhood [12–59 months]); and setting (research study vs programme evaluation at scale) [19].

## Methods

### Protocol development

Our protocol is based on the scoping review methods proposed by Arksey and O’Malley [2, 13]. This five stage process includes identifying the research question, identifying relevant studies, study selection, charting data and collating, summarising and reporting results. Recommendations of the Preferred Reporting Items for Systematic Reviews and Meta-Analyses Protocols (PRISMA-P) statement are provided [18] (Additional file 1). As this is a scoping review, we have not registered it with PROSPERO.

### Criteria for considering studies for inclusion

#### Types of studies

We will include all economic evaluations meeting the eligibility criteria regardless of whether they are conducted alongside an effectiveness intervention (e.g. a randomised controlled trial (RCT). This will include full economic evaluations (cost-effectiveness, cost-utility and cost-benefit analysis), cost analyses and comparative resource utilisation studies [11]. We will include published abstracts if there is sufficient information to allow us to assess study eligibility and risk of bias. If sufficient information is not available, the study will await assessment pending the publication of the full trial report or the provision of further information by trial authors.

#### Participants

All population groups will be included if they can be accessed by mainstream primary care providers. This will include pregnant women, families and their children aged under 5 years in high, middle and low income countries. Universal, targeted and high-risk groups will be included. Studies focused on mothers or children with specific disease entities such as malnutrition, HIV, autism and post partum depression will be excluded and considered as targeted high-risk groups.

#### Intervention

Interventions will include any non-medicinal intervention implemented in the presence (i.e. face to face, not in the waiting room) of a generalist primary care provider (health professional who is trained in clinical care, has a recognised clinical qualification and works at the first level of the health system, e.g. community health workers, Indigenous health workers, generalist nurses, health visitors, midwifes, child health nurses, general practitioners and other primary care doctors). These interventions will usually be motivational or educational in nature and use counselling skills. They may include anticipatory guidance, health promotion, health education, promotional interviewing, motivational interviewing, screening and surveillance. Delivery channels may include home visiting, child health checks, antenatal care, group programmes and telehealth. We will specifically exclude interventions that do not require the face to face presence of a primary care provider such as interventions provided in the waiting room (e.g videos and health promotion pamphlets). We will also exclude interventions that involve medicinal products such as nutritional supplementation, vaccinations and drug trials.

#### Control condition

Some studies will have a comparator group of other care or standard care. We are aware that control groups may vary substantially across studies. Thus, we will describe all control groups as carefully as we describe the intervention groups.

#### Outcomes

Our primary outcome measure will be cost per neurodevelopmental or cognitive health gain in children aged 0–4 years. This will often be reported as an incremental cost-effectiveness ratio. We will also include full details of all other cost measures. All measures of neurodevelopmental or cognitive function that have been previously validated as an appropriate test in this domain will be included. This will include general intelligence quotients and subscales including cognitive, language, speech, fine motor and gross motor development measured by standard tools such as the Bayley Scales of Infant and Toddler Development and the Griffiths Mental Development Scales. Measures of executive functioning and adaptive functioning will also be included. We will not include assessments of hearing or vision development. We will also exclude social and emotional development and child behavioural outcomes from this review.

### Search methods for identification of studies

#### Search strategy

The databases to be used for searching the relevant trials include the Cochrane Central Register of Controlled Trials (CENTRAL) (the Cochrane Library), MEDLINE (Ovid), EMBASE (Ovid) and Cochrane Database of Systematic Reviews, Health Technology Assessment (HTA) Database, NHS Economic Evaluation Database (NHS EED), Paediatric Economic Database Evaluation (PEDE) and Database of Abstracts of Reviews of Effects (DARE). An example of the MEDLINE search strategy is in Additional file 2. We will also search clinical trial registries such as Clinical-Trials.gov (http://clinicaltrials.gov/), International Standard Randomised Controlled Trial Number (ISRCTN) (http://www.controlled-trials.com), WHO International Clinical Trials Registry Platform (http://who.int/ictrp/en/) and UK Clinical Research Network Study Portfolio (http://www.nihr.ac.uk/research-and-impact/nihr-clinical-research-network-portfolio/). The search period will be from 2005 to 2016 (the most recent 10-year period) in all languages. Translation assistance will be sought.

#### Searching other sources

We will hand search reference lists from relevant articles chosen for potential inclusion in this review to identify further relevant studies. We will contact authors of included studies to determine whether there are any additional studies published, ongoing or unpublished that may be relevant. We will also search systematic review reference lists to identify any potentially relevant studies.

### Study selection

All titles and abstracts retrieved through the search strategy will be reviewed independently by two authors to identify studies that meet the inclusion criteria. Inclusion criteria at the title and abstract review level will be limited to any primary study reporting on primary care provider-related interventions to improve child cognition or neurodevelopment. Exclusion criteria at this stage will be qualitative and opinion articles and if the study clearly does not include children aged under 5 years or pregnant women.

Once articles have been identified, full-text articles will be retrieved and independently assessed by two independent review authors. We will specifically look for assessment of cost and cost effectiveness in the full-text articles and will exclude all studies that do not assess cost or cost-effectiveness. We will also assess articles for the exclusion criteria as listed above including medicinal interventions such as vaccination and nutritional supplementation and studies that only assess hearing or vision. If there is any disagreement, a third author will be asked to review the article. Authors will also be contacted for further clarification if necessary. We will document reasons for exclusion. Endnote X7 will be used throughout the process.

### Data extraction and management

Data will be collected by two independent reviewers using a standardised data collection form. Authors will be contacted for all missing data. Data collected from each study will include

- Basic descriptors (country in which study was conducted, year of publication, income level of country—high, middle and low)
- Population type—universal, targeted (including full details of the target group)
- Type of primary care provider—community health worker, Indigenous health worker, generalist nurse, health visitor, midwife, child health nurse, general practitioner, other primary care doctor
- Intervention type—anticipatory guidance, health promotion, health education, counselling, promotional interviewing, motivational interviewing, screening, surveillance, family partnership working
- Delivery channel—home visiting, group programmes, clinic visits, child health checks, antenatal care, telehealth, recall system, reminder systems, quality improvement initiatives
- Intervention implementation period—antenatal, infancy, early childhood
- Intervention frequency and duration
- Comparator group (e.g. full details of other care or standard care)
- Child neurodevelopmental outcomes—general intelligence quotients, subscales including cognitive, language, speech, fine motor, gross motor development, executive functioning, adaptive functioning
- Age of child at outcome measurement
- Study design
- Methods used to collect cost data (e.g. WHO CHOICE (Choosing Interventions that are Cost Effective), OECD (Organisation for Economic Co-operation and Development))
- Perspective of economic evaluation (societal, health service)
- Type of economic evaluation (e.g. cost-effectiveness)
- Other important economic analyses: sensitivity analyses, discounting
- Participant numbers in each group (numerators and denominators)

### Dealing with missing data

We will attempt to contact authors by email for any missing data.

### Data synthesis

Data will be reported in simple descriptive tables. We will also assess sub-groups descriptively: income level of country (high, middle and low); population type (universal vs targeted); time period when intervention was implemented (antenatal vs infancy [0–11 months] vs early childhood [12–59 months]); and setting (research study vs programme evaluation at scale). We will assess data using narrative approaches; no meta-analyses will be performed.

## Discussion

The findings of our review will be an important resource for policy makers and programmers in a variety of different settings. Our review will provide data about which studies and trials have assessed cost and cost-effectiveness of interventions to improve child neurodevelopment and the cost of implementing primary care provider programmes in high, middle and low income countries. We will provide up to date information on the impact of population type (universal vs targeted); time period when intervention was implemented (antenatal vs infancy [0–11 months] vs early childhood [12–59 months]); and the impact of setting (research study vs programme evaluation at scale) on cost and cost-effectiveness. In addition, some earlier cost-effectiveness studies and systematic reviews have reported very large clinical effect sizes raising a concern about publication bias [5]. Our scoping review will help to clarify this and other possible biases.

Our next steps include the assessment of other delivery channels that can improve child neurodevelopment such as electronic and social media (e.g. biofeedback, chat rooms, mass media, plain packaging, social marketing, websites, web boards and webinars) in high, middle and low income settings. Policy makers and programmers will then be able to compare and contrast our inventories and impacts. Our data will be able to be used as input parameters by modellers who need to calculate disability-adjusted life years (DALYs), quality-adjusted life years (QALYs), economic returns, benefit cost ratios, cost per health gain and burden of disease in a variety of different settings.

## Additional files

Additional file 1: PRISMA-P (Preferred Reporting Items for Systematic review and Meta-Analysis Protocols) 2015 checklist: recommended items to address in a systematic review protocol. (PDF 62 kb)
Additional file 2: Search strategy. (PDF 46 kb)
