# Supplementary material for: Economic evaluation of interventions delivered by primary care providers to improve neurodevelopment in children aged under 5 years: protocol for a scoping review
Source: Syst Rev. 2017 Mar 21;6:59. doi: 10.1186/s13643-017-0450-6 (PMC5359959; doi:10.1186/s13643-017-0450-6)
Supplement: Additional file 2: — Search strategy. (PDF 46 kb) [file 13643_2017_450_MOESM2_ESM.pdf]

## ADDITIONAL FILE 2 – SEARCH STRATEGY

MEDLINE (OVID)

| # | Searches |
|---|---|
| 1 | Economics/ |
| 2 | "costs and cost analysis"/ |
| 3 | Cost allocation/ |
| 4 | Cost-benefit analysis/ |
| 5 | Cost control/ |
| 6 | Cost of illness/ |
| 7 | Cost savings/ |
| 8 | Cost sharing/ |
| 9 | Medical savings accounts/ |
| 10 | Health care costs/ |
| 11 | Direct service costs/ |
| 12 | Employer health costs/ |
| 13 | Hospital costs/ |
| 14 | Health expenditures/ |
| 15 | Capital expenditures/ |
| 16 | Value of life/ |
| 17 | exp economics, hospital/ |
| 18 | exp economics, medical/ |
| 19 | Economics, nursing/ |
| 20 | exp "fees and charges"/ |
| 21 | exp budgets/ |
| 22 | (low adj cost).mp. |
| 23 | (high adj cost).mp. |
| 24 | (health?care adj cost$).mp. |
| 25 | (fiscal or funding or financial or finance).tw. |
| 26 | (cost adj estimate$).mp. |
| 27 | (cost adj variable).mp. |
| 28 | (unit adj cost$).mp. |
| 29 | cost$.mp. |
| 30 | OR 1-29 |
| 31 | exp Child/ |
| 32 | exp Infant/ |
| 33 | (child$ or infan$ or baby or babies or preschool$ or pre-school$).tw. |
| 34 | OR 31-33 |
| 35 | exp Pregnancy/ |
| 36 | exp Maternal Health Services/ |
| 37 | exp Postpartum Period/ |
| 38 | antepart$.mp. |
| 39 | pregnan$.mp. |

| | |
|---|---|
| 40 | prenatal$.mp. |
| 41 | antenatal$.mp. |
| 42 | perinatal$.mp. |
| 43 | postnatal$.mp. |
| 44 | postpart$.mp. |
| 45 | OR 35-44 |
| 46 | Patient Education as Topic/ |
| 47 | exp health education/ |
| 48 | patient care.ti,ab. |
| 49 | (patient? adj3 (educat$ or counsel$ or helping or advising or directing or caring or assist? or assisting or assistance or teaching or learning or promoting)).ti,ab. |
| 50 | (health adj2 (improv$ or promotion)).ti,ab. |
| 51 | exp health promotion/ |
| 52 | public health/ |
| 53 | preventive medicine/ |
| 54 | primary prevention/ |
| 55 | "Early Intervention (Education)"/ |
| 56 | exp Preventive Health Services/ |
| 57 | Early Medical Intervention/ |
| 58 | Consumer Health Information/ |
| 59 | Health Literacy/ |
| 60 | mass screening/ |
| 61 | medical history taking/ |
| 62 | population surveillence/ |
| 63 | early diagnosis/ |
| 64 | (monitor$ or screen$ or surveillance or assessment$).tw. |
| 65 | case finding.tw. |
| 66 | exp community health services/ |
| 67 | house call/ |
| 68 | (((home* or in-home* or at-home*) adj3 nurs*) not nursing next home*).mp. [mp=title, abstract, original title, name of substance word, subject heading word, keyword heading word, protocol supplementary concept word, rare disease supplementary concept word, unique identifier] |
| 69 | ((home* or in-home* or at-home* or house* or domicil* or communit* or neighborhood* or neighbourhood*) adj5 (visit* or support* or program* or intervention*)).mp. [mp=title, abstract, original title, name of substance word, subject heading word, keyword heading word, protocol supplementary concept word, rare disease supplementary concept word, unique identifier] |
| 70 | anticipatory guidance.mp. |
| 71 | primary care/ |
| 72 | motivational interviewing.mp. or exp Motivational Interviewing/ |
| 73 | exp child health services/ or exp community health nursing/ |
| 74 | general health check.mp. |
| 75 | counsel$.mp. |
| 76 | exp Counseling/ec, ed [Economics, Education] |
| 77 | exp child development/ |

| | |
|---|---|
| 78 | (development$ adj2 (delay$ or deviat$ or disabilit$ or disorder$ or milestone$ or risk$)).tw. |
| 79 | language development disorders/ |
| 80 | speech disorders/ |
| 81 | ((speech or language) adj2 (delay$ or disorder$)).tw. |
| 82 | communication disorders/ |
| 83 | (communication adj2 (delay$ or disorder$)).tw. |
| 84 | Motor Skills/ |
| 85 | ((motor or psychomotor) adj2 (delay$ or disabilit$ or disorder$)).tw. |
| 86 | adaptive behavior/ |
| 87 | (cogniti$ adj2 (delay$ or deviat$ or disabilit$ or disorder$ or milestone$ or risk$)).mp. |
| 88 | exp adaptation, psychological/ or exp child development/ |
| 89 | OR 46-76 |
| 90 | OR 77-88 |
| 91 | 34 or 45 |
| 92 | 30 and 89 and 90 and 91 |
| 93 | limit 92 to yr="2005 -Current" |
